# 4,4′-({[(Pyridine-2,6-di­yl)bis­(methyl­ene)]bis­(­oxy)}bis­(methyl­ene))dibenzo­nitrile

**DOI:** 10.1107/S1600536814000245

**Published:** 2014-01-11

**Authors:** Bohari M. Yamin, Nurul A. Kamalulazmy, Nurul I. Hassan

**Affiliations:** a School of Chemical Sciences and Food Technology, Universiti Kebangsaan Malaysia, 43600 Bangi, Selangor, Malaysia

## Abstract

The complete title molecule, C_23_H_19_N_3_O_2_, is generated by a twofold axis passing through the central ring. The two oxymethyl­benzo­nitrile arms are attached at the *meta* positions of the central pyridine ring. The dihedral angle between the pyridine ring and benzene ring of both arms is 84.55 (6)° while the benzene rings make a dihedral angle of 46.07 (7)°. In the crystal, weak C—H⋯π inter­actions link the molecules sheets parallel to the *ac* plane.

## Related literature   

For bond-length data, see: Allen *et al.* (1987 ▶). For related structures, see: Lima *et al.* (2011 ▶); Wang & Zhao (2008 ▶): Zhao (2008 ▶); Xiao & Zhao (2008 ▶).
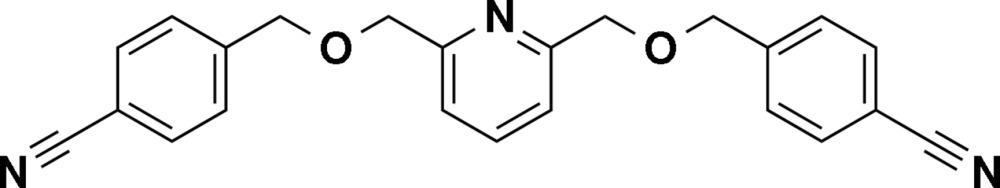


## Experimental   

### 

#### Crystal data  

C_23_H_19_N_3_O_2_
*M* *_r_* = 369.41
Monoclinic, 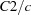

*a* = 14.1838 (7) Å
*b* = 7.5493 (4) Å
*c* = 18.4619 (12) Å
β = 107.837 (2)°
*V* = 1881.83 (18) Å^3^
*Z* = 4
Mo *K*α radiation
μ = 0.09 mm^−1^
*T* = 296 K
0.49 × 0.44 × 0.34 mm

#### Data collection  

Bruker SMART APEX CCD area-detector diffractometer
Absorption correction: multi-scan (*SADABS*; Bruker, 2009 ▶) *T*_min_ = 0.960, *T*_max_ = 0.972
21611 measured reflections
1846 independent reflections
1519 reflections with *I* > 2σ(*I*)
*R*_int_ = 0.040

#### Refinement  

*R*[*F* ^2^ > 2σ(*F* ^2^)] = 0.039
*wR*(*F* ^2^) = 0.106
*S* = 1.05
1846 reflections
129 parameters
H-atom parameters constrained
Δρ_max_ = 0.10 e Å^−3^
Δρ_min_ = −0.12 e Å^−3^

### 

Data collection: *SMART* (Bruker, 2009 ▶); cell refinement: *SAINT* (Bruker, 2009 ▶); data reduction: *SAINT*; program(s) used to solve structure: *SHELXS97* (Sheldrick, 2008 ▶); program(s) used to refine structure: *SHELXL97* (Sheldrick, 2008 ▶); molecular graphics: *SHELXTL* (Sheldrick, 2008 ▶); software used to prepare material for publication: *SHELXTL* and *PLATON* (Spek, 2009 ▶).

## Supplementary Material

Crystal structure: contains datablock(s) global, I. DOI: 10.1107/S1600536814000245/hg5373sup1.cif

Structure factors: contains datablock(s) I. DOI: 10.1107/S1600536814000245/hg5373Isup2.hkl

Supporting information file. DOI: 10.1107/S1600536814000245/hg5373Isup3.cml

CCDC reference:

Additional supporting information:  crystallographic information; 3D view; checkCIF report

## Figures and Tables

**Table 1:** Hydrogen-bond geometry (Å, °) *Cg* is the centroid of the N1/C1–C3/C2′/C3′ ring.

*D*—H⋯*A*	*D*—H	H⋯*A*	*D*⋯*A*	*D*—H⋯*A*
C8—H8⋯*Cg* ^i^	0.93	2.87	3.7180 (15)	152

Symmetry code: (i) 
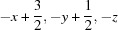
.

**Fig. 1.**
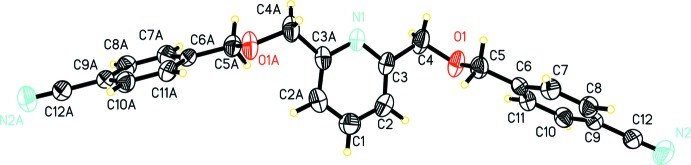
: Molecular structure of (I) with 50% probability displacement ellipsoids. Symmetry code A: (-x + 2, y, -z + 1/2).

## supplementary crystallographic information

### 1. Comment

Despite the wide application of dibenzonitrile compounds in the area of chemical synthesis and industrial, the structural study of dibenzonitrile derivatives are less reported. Thiadiazole dibenzonitrile (Wang & Zhao, 2008) and naphthalene dibenzonitrile (Lima *et al.*, 2011) are some examples of dibenzonitrile derivatives. On the other hand, 4,4'-{[1,1'-Methylenebis (naphthalene-2,1-diyl)]bis(oxymethylene)}dibenzonitrile (Zhao, 2008) and 4,4'-(Oxydimethylene)dibenzonitrile (Xiao & Zhao, 2008) are few examples of bis(oxymethylene)dibenzonitriles. The title compound is a bis(methylene)bis(oxy) bis(methylene)dibenzonitrile in which the oxymtheylenedibenzonitrile arms are connected by 2,6-pyridine linkager at *meta* position (Figure 1) making the whole molecule, a bird like structure. The dihedral angle between the two benzene rings [(C6/C7/C8/C9/C10/C11), (C6a/C7a/C8a/C9a/C10*a*/C11*a*)] and the central pyridine (C1/C2/C3/C2a/C3a/N1) is 84.55 (6)°. The maximum deviation of the two planes is 0.005 (1) Å for C2 atom from the least square plane of the pyridine ring. The bond lengths are in normal ranges (Allen *et al.*,1987). In the structure, no intermolecular hydrogen bonds were observed except the presence of C8—H8···π bonds with the pyridine ring (H8—centroid *Cg* distance of 2.87 Å, and *X*—H···centroid angle = 152°).

### 2. Experimental

NaH (10.72 g, 17.8 mmol) was carefully added to a solution of 2,6-pyridine dimethanol (1.0 g, 7.2 mmol) in dry THF (35 ml). The resulting mixture was refluxed for 1.5 h and allowed to cool to room temperature. Next, 4-bromomethyl benzonitrile (3.0 g, 15.3 mmol) was added and continued to reflux for another 24 h at 75°C. Water was slowly added to quench the reaction. The organic layers were extracted into ethyl acetate. The combined organic layers were dried over magnesium sulfate, filtered and concentrated under reduced pressure. The resulting residue was purified using column chromatography (Hexane:Ethyl acetate) to furnish a colorless powder. Single crystals were obtained from the solution of Hexane:Ethyl acetate after one day of evaporation (yield 87%, m.p 378.8–380 K). ^1^H NMR (300.1 MHz, CDCl_3_): 7.75 (1*H*, t, 3J_HH_ 7.7 Hz; ArH), 7.65 (4*H*, d, 3J_HH_ 8.4 Hz;4 *x* ArH),7.49 (4*H*, d, 3J_HH_ 8.6 Hz; 4 *x* ArH), 7.40 (2*H*, d, 3J_HH_ 7.8 Hz; 2 *x* ArH), 4.71 (4*H*, s; 2 *x* CH_2_), 4.70 (4*H*, s; 2 *x* CH_2_);^13^C NMR (75.5 MHz, CDCl_3_):*C* 157.6 (ArC), 143.6 (ArC), 137.6 (ArCH), 132.4 (ArCH), 127.9 (ArCH), 120.4 (ArCH), 118.9 (CN), 111.6 (ArC),73.7 (CH_2_), 72.1 (CH_2_); MS (CI^+^) m/*z* 239.0 (27), 370.2 ([*M*+H]^+^, 100); HRMS (CI^+^) m/*z* calculated for C_23_H_20_N_3_O_2_ [*M*+H]^+^ 370.1556, found 370.1563.

### 3. Refinement

After their location in the difference map, the H-atoms attached to the C and N atoms were fixed geometrically at ideal positions and allowed to ride on the parent atoms with C—H = 0.93 Å, with *U*_iso_(H)=1.2*U*_eq_(C).

### Crystal data

**Table:**

C_23_H_19_N_3_O_2_	*F*(000) = 776
*M**_r_* = 369.41	*D*_x_ = 1.304 Mg m^−^^3^
Monoclinic, *C*2/*c*	Mo *K*α radiation, λ = 0.71073 Å
Hall symbol: -C 2yc	Cell parameters from 8925 reflections
*a* = 14.1838 (7) Å	θ = 3.0–26.0°
*b* = 7.5493 (4) Å	µ = 0.09 mm^−^^1^
*c* = 18.4619 (12) Å	*T* = 296 K
β = 107.837 (2)°	Block, colourless
*V* = 1881.83 (18) Å^3^	0.49 × 0.44 × 0.34 mm
*Z* = 4	

### Data collection

**Table:**

Bruker SMART APEX CCD area-detector diffractometer	1846 independent reflections
Radiation source: fine-focus sealed tube	1519 reflections with *I* > 2σ(*I*)
Graphite monochromator	*R*_int_ = 0.040
Detector resolution: 83.66 pixels mm^-1^	θ_max_ = 26.0°, θ_min_ = 3.1°
ω scan	*h* = −17→17
Absorption correction: multi-scan (*SADABS*; Bruker, 2009)	*k* = −9→9
*T*_min_ = 0.960, *T*_max_ = 0.972	*l* = −22→22
21611 measured reflections	

### Refinement

**Table:**

Refinement on *F*^2^	Secondary atom site location: difference Fourier map
Least-squares matrix: full	Hydrogen site location: inferred from neighbouring sites
*R*[*F*^2^ > 2σ(*F*^2^)] = 0.039	H-atom parameters constrained
*wR*(*F*^2^) = 0.106	*w* = 1/[σ^2^(*F*_o_^2^) + (0.0485*P*)^2^ + 0.9538*P*] where *P* = (*F*_o_^2^ + 2*F*_c_^2^)/3
*S* = 1.05	(Δ/σ)_max_ < 0.001
1846 reflections	Δρ_max_ = 0.10 e Å^−^^3^
129 parameters	Δρ_min_ = −0.12 e Å^−^^3^
0 restraints	Extinction correction: *SHELXL97* (Sheldrick, 2008), Fc^*^=kFc[1+0.001xFc^2^λ^3^/sin(2θ)]^-1/4^
Primary atom site location: structure-invariant direct methods	Extinction coefficient: 0.0196 (14)

### Special details

**Table:**

Geometry. All e.s.d.'s (except the e.s.d. in the dihedral angle between two l.s. planes) are estimated using the full covariance matrix. The cell e.s.d.'s are taken into account individually in the estimation of e.s.d.'s in distances, angles and torsion angles; correlations between e.s.d.'s in cell parameters are only used when they are defined by crystal symmetry. An approximate (isotropic) treatment of cell e.s.d.'s is used for estimating e.s.d.'s involving l.s. planes.
Refinement. Refinement of *F*^2^ against ALL reflections. The weighted *R*-factor *wR* and goodness of fit *S* are based on *F*^2^, conventional *R*-factors *R* are based on *F*, with *F* set to zero for negative *F*^2^. The threshold expression of *F*^2^ > σ(*F*^2^) is used only for calculating *R*-factors(gt) *etc*. and is not relevant to the choice of reflections for refinement. *R*-factors based on *F*^2^ are statistically about twice as large as those based on *F*, and *R*- factors based on ALL data will be even larger.

### Fractional atomic coordinates and isotropic or equivalent isotropic displacement parameters (Å^2^)

**Table:**

	*x*	*y*	*z*	*U*_iso_*/*U*_eq_	
O1	0.73355 (7)	0.39554 (16)	0.18488 (6)	0.0547 (3)	
N1	1.0000	0.4615 (2)	0.2500	0.0416 (4)	
N2	0.25164 (10)	0.0851 (2)	−0.08574 (9)	0.0706 (5)	
C1	1.0000	0.0946 (3)	0.2500	0.0530 (6)	
H1	1.0000	−0.0286	0.2500	0.064*	
C2	0.91206 (10)	0.1866 (2)	0.23419 (8)	0.0495 (4)	
H2	0.8519	0.1269	0.2230	0.059*	
C3	0.91523 (9)	0.3697 (2)	0.23533 (7)	0.0415 (4)	
C4	0.82379 (10)	0.4813 (2)	0.22471 (10)	0.0560 (4)	
H4A	0.8206	0.5169	0.2744	0.067*	
H4B	0.8300	0.5880	0.1973	0.067*	
C5	0.71745 (10)	0.3882 (2)	0.10575 (8)	0.0463 (4)	
H5A	0.7659	0.3102	0.0951	0.056*	
H5B	0.7257	0.5054	0.0870	0.056*	
C6	0.61510 (9)	0.32139 (17)	0.06575 (8)	0.0369 (3)	
C7	0.58164 (10)	0.3218 (2)	−0.01320 (8)	0.0429 (4)	
H7	0.6225	0.3645	−0.0402	0.052*	
C8	0.48871 (11)	0.2599 (2)	−0.05204 (8)	0.0452 (4)	
H8	0.4672	0.2599	−0.1050	0.054*	
C9	0.42703 (10)	0.19725 (18)	−0.01217 (8)	0.0403 (3)	
C10	0.45942 (10)	0.19748 (19)	0.06662 (8)	0.0443 (4)	
H10	0.4182	0.1562	0.0936	0.053*	
C11	0.55300 (10)	0.25913 (19)	0.10512 (8)	0.0422 (4)	
H11	0.5746	0.2588	0.1580	0.051*	
C12	0.32950 (11)	0.1331 (2)	−0.05269 (9)	0.0496 (4)	

### Atomic displacement parameters (Å^2^)

**Table:**

	*U*^11^	*U*^22^	*U*^33^	*U*^12^	*U*^13^	*U*^23^
O1	0.0282 (5)	0.0835 (9)	0.0491 (6)	−0.0047 (5)	0.0069 (4)	−0.0156 (5)
N1	0.0293 (8)	0.0550 (10)	0.0369 (8)	0.000	0.0050 (6)	0.000
N2	0.0447 (8)	0.0701 (10)	0.0859 (11)	−0.0081 (7)	0.0035 (7)	−0.0136 (8)
C1	0.0554 (13)	0.0502 (13)	0.0511 (12)	0.000	0.0130 (10)	0.000
C2	0.0393 (8)	0.0624 (10)	0.0446 (8)	−0.0113 (7)	0.0095 (6)	−0.0047 (7)
C3	0.0302 (7)	0.0592 (9)	0.0329 (7)	−0.0029 (6)	0.0063 (5)	−0.0071 (6)
C4	0.0297 (7)	0.0736 (11)	0.0599 (9)	−0.0005 (7)	0.0065 (6)	−0.0220 (8)
C5	0.0327 (7)	0.0542 (9)	0.0498 (8)	0.0018 (6)	0.0095 (6)	−0.0016 (7)
C6	0.0302 (6)	0.0342 (7)	0.0442 (7)	0.0066 (5)	0.0084 (5)	−0.0003 (6)
C7	0.0403 (7)	0.0467 (8)	0.0441 (8)	0.0014 (6)	0.0164 (6)	−0.0001 (6)
C8	0.0469 (8)	0.0485 (8)	0.0368 (7)	0.0028 (7)	0.0080 (6)	−0.0030 (6)
C9	0.0343 (7)	0.0344 (7)	0.0478 (8)	0.0030 (5)	0.0061 (6)	−0.0013 (6)
C10	0.0368 (7)	0.0469 (8)	0.0500 (8)	0.0009 (6)	0.0142 (6)	0.0073 (6)
C11	0.0381 (7)	0.0480 (8)	0.0376 (7)	0.0046 (6)	0.0071 (6)	0.0040 (6)
C12	0.0419 (8)	0.0432 (8)	0.0588 (9)	0.0025 (7)	0.0080 (7)	−0.0039 (7)

### Geometric parameters (Å, º)

**Table:**

O1—C5	1.4088 (18)	C5—H5A	0.9700
O1—C4	1.4217 (17)	C5—H5B	0.9700
N1—C3^i^	1.3417 (16)	C6—C11	1.3846 (19)
N1—C3	1.3417 (16)	C6—C7	1.3879 (19)
N2—C12	1.1449 (19)	C7—C8	1.3751 (19)
C1—C2	1.3786 (19)	C7—H7	0.9300
C1—C2^i^	1.3786 (19)	C8—C9	1.387 (2)
C1—H1	0.9300	C8—H8	0.9300
C2—C3	1.382 (2)	C9—C10	1.3847 (19)
C2—H2	0.9300	C9—C12	1.4400 (19)
C3—C4	1.508 (2)	C10—C11	1.3804 (19)
C4—H4A	0.9700	C10—H10	0.9300
C4—H4B	0.9700	C11—H11	0.9300
C5—C6	1.5001 (18)		
			
C5—O1—C4	112.87 (12)	C6—C5—H5B	109.6
C3^i^—N1—C3	117.77 (18)	H5A—C5—H5B	108.1
C2—C1—C2^i^	119.5 (2)	C11—C6—C7	118.96 (12)
C2—C1—H1	120.3	C11—C6—C5	122.07 (12)
C2^i^—C1—H1	120.3	C7—C6—C5	118.97 (12)
C1—C2—C3	118.50 (14)	C8—C7—C6	120.77 (13)
C1—C2—H2	120.8	C8—C7—H7	119.6
C3—C2—H2	120.8	C6—C7—H7	119.6
N1—C3—C2	122.86 (14)	C7—C8—C9	119.92 (13)
N1—C3—C4	114.79 (14)	C7—C8—H8	120.0
C2—C3—C4	122.25 (13)	C9—C8—H8	120.0
O1—C4—C3	114.53 (13)	C10—C9—C8	119.77 (12)
O1—C4—H4A	108.6	C10—C9—C12	120.20 (13)
C3—C4—H4A	108.6	C8—C9—C12	120.03 (13)
O1—C4—H4B	108.6	C11—C10—C9	119.91 (13)
C3—C4—H4B	108.6	C11—C10—H10	120.0
H4A—C4—H4B	107.6	C9—C10—H10	120.0
O1—C5—C6	110.41 (11)	C10—C11—C6	120.67 (13)
O1—C5—H5A	109.6	C10—C11—H11	119.7
C6—C5—H5A	109.6	C6—C11—H11	119.7
O1—C5—H5B	109.6	N2—C12—C9	178.67 (19)
			
C2^i^—C1—C2—C3	−0.50 (9)	C11—C6—C7—C8	−0.7 (2)
C3^i^—N1—C3—C2	−0.54 (10)	C5—C6—C7—C8	179.27 (13)
C3^i^—N1—C3—C4	175.99 (14)	C6—C7—C8—C9	0.5 (2)
C1—C2—C3—N1	1.1 (2)	C7—C8—C9—C10	0.0 (2)
C1—C2—C3—C4	−175.22 (11)	C7—C8—C9—C12	179.68 (14)
C5—O1—C4—C3	−76.99 (18)	C8—C9—C10—C11	−0.3 (2)
N1—C3—C4—O1	159.71 (12)	C12—C9—C10—C11	180.00 (13)
C2—C3—C4—O1	−23.7 (2)	C9—C10—C11—C6	0.1 (2)
C4—O1—C5—C6	−171.60 (12)	C7—C6—C11—C10	0.3 (2)
O1—C5—C6—C11	−6.58 (19)	C5—C6—C11—C10	−179.59 (13)
O1—C5—C6—C7	173.49 (12)		

Symmetry code: (i) −*x*+2, *y*, −*z*+1/2.

### Hydrogen-bond geometry (Å, º)

Cg is the centroid of the N1/C1–C3/C2'/C3' ring.

**Table:**

*D*—H···*A*	*D*—H	H···*A*	*D*···*A*	*D*—H···*A*
C11—H11···O1	0.93	2.39	2.735 (2)	102
C8—H8···*Cg*^ii^	0.93	2.87	3.7180 (15)	152

Symmetry code: (ii) −*x*+3/2, −*y*+1/2, −*z*.
